# Clinical efficacy and predictive factors of photodynamic therapy combined with recombinant mussel mucin repair dressing in treating post-inflammatory hyperpigmentation

**DOI:** 10.3389/fmed.2025.1654474

**Published:** 2026-01-29

**Authors:** Jianjun Nie, Zengmiao Hou, Peimei Zhou, Ya Zhang, Yonghong Lu

**Affiliations:** 1 Chengdu Second People's Hospital, Chengdu, China; 2 Xi an DeNovo Hith Medical Technology Co, Ltd, Xian, China; 3 The Eighth Affiliated Hospital of Sun Yat-sen University, Shenzhen, China

**Keywords:** post-inflammatory hyperpigmentation, photodynamic therapy, mussel mucin, repair dressing, machine learning, precision dermatology, melanogenesis, antioxidant therapy

## Abstract

**Objective:**

To evaluate the efficacy and safety of photodynamic therapy (PDT) combined with recombinant mussel mucin repair dressing in treating post-inflammatory hyperpigmentation (PIH), and to assess its impact on patient quality of life.

**Methods:**

In a prospective, controlled, non-randomized clinical study, we enrolled 310 patients (Fitzpatrick III–IV) with epidermal or dermal PIH. Patients underwent PDT by wavelength and energy protocol, followed by immediate application of recombinant mussel mucin repair dressing. Pigmentation area, melanin density, adverse events, recurrence, and quality of life were assessed over 14 months.

**Results:**

The combined therapy achieved significantly greater pigment clearance at 4 weeks (42.3 vs. 25.6% in controls, *p* < 0.001), larger melanin density reduction (31.5 vs. 17.2%, *p* = 0.002), fewer phototoxic reactions (6.5 vs. 14.1%, *p* = 0.021), and lower recurrence at 14 months (13.2 vs. 27.8%, *p* = 0.011). Patient-reported quality of life markedly improved (median DLQI score from 35 to 68, *p* < 0.001).

**Conclusion:**

PDT combined with recombinant mussel mucin repair dressing provides superior pigment clearance, reduced recurrence, improved safety, and enhanced quality of life compared with standard therapy. This multimodal approach offers a promising option for PIH management in darker skin types.

## Introduction

Post-inflammatory hyperpigmentation (PIH) is a prevalent acquired pigmentary disorder, particularly common in individuals with darker skin types such as Fitzpatrick III–VI, where it causes significant cosmetic and psychological distress (1, 2). PIH often results from common skin insults—including acne, eczema, trauma, and cosmetic interventions—and its prevalence can reach up to 65% in affected populations, especially among Asians and Africans (1, 3, 4). Beyond its cosmetic impact, PIH exerts a substantial burden on patients' quality of life, negatively affecting self-esteem and contributing to anxiety or depression (5).

The pathogenesis of PIH is multifactorial. Cutaneous inflammation triggers the release of pro-inflammatory cytokines, such as TNF-α and IL-6, which stimulate melanocyte proliferation and increase the expression of melanogenic enzymes like tyrosinase (6, 7). This cascade leads to excessive melanin production and deposition in both epidermal and dermal layers, especially in sites of skin injury or persistent irritation (8, 9). Disruption of the epidermal barrier further amplifies these effects, increasing transepidermal water loss and facilitating ongoing inflammation and abnormal melanogenesis (10, 11). Recent evidence highlights the additional role of oxidative stress in maintaining a microenvironment that favors melanin overproduction and persistent pigmentation (12, 13).

Traditional approaches to PIH management include topical depigmenting agents (hydroquinone, retinoids, azelaic acid), chemical peels, and various energy-based devices such as Q-switched lasers and fractional resurfacing (14–16). While topical hydroquinone remains a first-line therapy, it is associated with adverse effects such as irritant dermatitis and, rarely, exogenous ochronosis, especially in patients with darker skin (15). Chemical peels and retinoids offer incremental improvements but may trigger irritation or worsen pigmentation in sensitive individuals (17). Energy-based treatments, though effective for some, are limited by the risk of post-procedure inflammation, secondary PIH, and high relapse rates, particularly in patients of color (16, 18). These limitations have fueled interest in novel strategies that target both the underlying inflammation and barrier dysfunction implicated in PIH (10, 19).

Growing research demonstrates that restoration of epidermal barrier function is crucial for effective PIH management (10, 20). Interventions that support the repair of the stratum corneum, decrease transepidermal water loss, and reduce ongoing inflammation contribute to improved pigmentary outcomes and decreased recurrence (11, 20). Concurrently, antioxidant therapies are being explored for their potential to reduce oxidative stress, disrupt pro-melanogenic pathways, and complement established depigmenting regimens (12, 21).

Photodynamic therapy (PDT) has emerged as a promising modality for pigmentary disorders, including PIH. PDT involves the activation of a photosensitizing agent with specific wavelengths of light, producing reactive oxygen species that can selectively target pigmented or inflamed tissue (22, 23). PDT's ability to induce controlled injury, modulate inflammatory mediators, and promote tissue remodeling underlies its utility in recalcitrant pigmentation. However, PDT is not without risk: phototoxic reactions, transient barrier compromise, and variable pigment clearance necessitate careful post-treatment care and individualized protocols, especially in patients with higher melanin content (23, 24).

Recent advances in biomaterial science have led to the development of bioinspired wound dressings and skin barrier repair systems. Among these, mussel-inspired adhesive proteins and hydrogels have attracted significant interest due to their strong tissue adhesion, robust biocompatibility, antioxidant activity, and support for cellular regeneration (25, 26). Recombinant mussel protein hydrogels mimic the natural defense mechanisms of marine mussels, providing both immediate physical barrier restoration and sustained drug or antioxidant delivery to damaged skin (27, 28). Their unique polyphenolic chemistry enables potent free-radical scavenging and anti-inflammatory effects, which are especially beneficial in the context of post-inflammatory pigmentary disorders (29).

The integration of photodynamic therapy with advanced barrier-restoring dressings—such as recombinant mussel mucin—represents a novel paradigm for PIH management. Immediately after PDT, application of such dressings can mitigate phototoxicity, accelerate epidermal healing, dampen inflammatory cascades, and reduce oxidative damage, all while maintaining a moist and protective microenvironment for skin repair (24, 27, 30). Laboratory and preclinical studies confirm these dressings' ability to inhibit tyrosinase, suppress melanin synthesis, and promote keratinocyte proliferation, supporting their mechanistic role in pigmentation control (28, 29, 31).

At the same time, rapid advances in artificial intelligence (AI) and machine learning (ML) are transforming clinical dermatology. ML algorithms can integrate and analyze multidimensional clinical, demographic, and laboratory data to identify predictive features and forecast treatment response in pigmentary disorders (32, 33). Compared to conventional statistical approaches, ensemble ML models—such as random forest, gradient boosting, and voting regressors—demonstrate superior accuracy for predicting pigmentation reduction, risk of recurrence, and patient outcomes, especially when trained on large, well-curated datasets (33, 34). The adoption of these tools enables personalized risk stratification and data-driven optimization of PIH therapies (34, 35).

Despite these advances, few studies have systematically explored the combined efficacy of PDT and recombinant mussel mucin dressing in PIH, or leveraged machine learning to interrogate their clinical and mechanistic effects in real-world populations. To address this gap, we conducted a prospective observational study involving 310 patients, integrating *in vitro* and *ex vivo* analysis to evaluate the therapeutic role of recombinant mussel mucin dressing in PIH management, integrating dynamic imaging, *in vitro* and *in vivo* laboratory assays, and robust data science methods. Our aim was to evaluate the short- and long-term efficacy, safety, mechanistic effects, and predictors of response for combined PDT and mussel mucin repair therapy in a diverse Asian cohort. This multidimensional investigation provides new insights into barrier-restoring strategies for PIH, while demonstrating the utility of machine learning for precision dermatology and personalized pigment management.

## Materials and methods

### Study population and inclusion criteria

This perspective, controlled, non randomized clinical study was conducted at Chengdu Second People's Hospital from 2022 to 2024. A total of 310 adult patients (aged 18–55) with confirmed post-inflammatory hyperpigmentation (PIH) were enrolled. Participants predominantly consisted of females (~70%) and represented Asian Fitzpatrick skin types III–IV (medium to dark skin). Eligible subjects had a disease course of at least 3 months with a stable lesion phase (no new lesions in the preceding month), a VISIA-predicted macular area ≥5 cm^2^, and a melanin optical density (OD) value ≥0.25 as determined by skin melanometry. Approximately 60% of patients presented with epidermal PIH and 40% with dermal PIH. The primary etiologies included acne (45%), laser surgery (30%), and trauma (25%). Inclusion criteria required clinical diagnosis of epidermal or dermal PIH via dermatoscopy and confocal microscopy, age 18–55, BMI 18.5–28 kg/m^2^, stable pigmentation for at least 3 months, melanin density >30% higher than adjacent normal skin, willingness and ability to complete a 6-month follow-up, absence of photosensitivity (confirmed by negative serum porphyrin), and normal hepatic and renal function (ALT ≤ 40 U/L, Cr ≤ 1.2 mg/dl). Patients were excluded if they were pregnant or breastfeeding, prone to keloid formation, had active skin infections, had a history of photosensitive disorders, or demonstrated psychiatric illness or poor compliance. All participants provided written informed consent prior to enrollment. Baseline clinical and demographic characteristics are summarized in Table 1.

**Table 1:** Baseline demographic and clinical characteristics of enrolled patients and distribution by treatment group.

**Variable**	**Epidermal 80 J/cm^2^ (*n* = 60)**	**Epidermal 100 J/cm^2^ (*n* = 60)**	**Dermal 80 J/cm^2^ (*n* = 60)**	**Dermal 100 J/cm^2^ (*n* = 60)**	**Laser control (*n* = 70)**	***p*-value**
Age, years (mean ± SD)	29.8 ± 8.3	30.1 ± 8.1	30.5 ± 7.9	29.7 ± 7.6	29.9 ± 8.2	0.84
Gender (F/M), *n* (%)	42/18 (70/30)	43/17 (71.7/28.3)	41/19 (68.3/31.7)	44/16 (73.3/26.7)	48/22 (68.6/31.4)	0.97
Fitzpatrick skin type (III/IV)	36/24 (60/40)	35/25 (58.3/41.7)	38/22 (63.3/36.7)	37/23 (61.7/38.3)	43/27 (61.4/38.6)	0.93
Lesion duration (months, mean ± SD)	9.2 ± 3.8	9.5 ± 3.7	9.3 ± 3.5	9.4 ± 3.4	9.1 ± 3.9	0.86
Baseline pigmentation area (cm^2^)	6.1 ± 1.2	6.2 ± 1.1	6.1 ± 1.3	6.2 ± 1.0	6.2 ± 1.2	0.99
Baseline melanin density (OD)	0.29 ± 0.05	0.28 ± 0.06	0.29 ± 0.04	0.28 ± 0.05	0.29 ± 0.06	0.91
PIH etiology (acne/surgery/trauma)	27/18/15	28/19/13	26/19/15	27/18/15	31/22/17	0.89

### Application of recombinant mussel mucin membrane

The recombinant mussel mucin membrane used in this study was produced through recombinant protein expression in *E. coli*. The membrane was fabricated by purifying the mussel mucin protein (derived from *Mytilus edulis*), which was expressed and then processed into a membrane form. The membrane's structural integrity was assessed using SEM.

### Experimental procedures and instrumentation

Photodynamic therapy (PDT) has emerged as a promising modality for pigmentary disorders, including PIH. PDT involves the activation of a photosensitizing agent with specific wavelengths of light, producing reactive oxygen species that can selectively target pigmented or inflamed tissue (22). The selection of PDT parameters is crucial for treatment efficacy. For epidermal PIH, 630 nm wavelength achieves optimal penetration depth (1–2 mm) and activates protoporphyrin IX while maintaining selectivity for melanin (23). For dermal PIH, 595 nm wavelength enables deeper tissue penetration (2–3 mm), reduces melanin absorption, and effectively targets deep melanin deposits. Energy density selection balances efficacy and safety: 80 J/cm^2^ can reduce phototoxic reactions in darker skin, while 100 J/cm^2^ achieves superior pigment clearance (42.3% area reduction vs. 38.5%) with a manageable safety margin. The combination of wavelength-specific targeting and optimized energy density creates a synergistic effect for comprehensive PIH management (24).

Each patient was assigned to one of five groups: PDT at 630 nm (epidermal PIH, 80 or 100 J/cm^2^), PDT at 595 nm (dermal PIH, 80 or 100 J/cm^2^), or a standard laser therapy control group. Patients were randomized into five treatment groups based on PIH depth and PDT parameters. Group allocation was determined by the combination of wavelength selection (630 nm for epidermal PIH, 595 nm for dermal PIH) and energy density (80 J/cm^2^ or 100 J/cm^2^). The treatment groups were: Epidermal 80 J/cm^2^ (*n* = 60), Epidermal 100 J/cm^2^ (*n* = 60), Dermal 80 J/cm^2^ (*n* = 60), Dermal 100 J/cm^2^ (*n* = 60), and Laser Control (*n* =70). Each group received standardized PDT treatment using the Omnilux Revive device with specific wavelength and energy density parameters. The experimental groups (Epidermal 80 J/cm^2^, Epidermal 100 J/cm^2^, Dermal 80 J/cm^2^, Dermal 100 J/cm^2^) received combined PDT and recombinant mussel mucin repair dressing (manufactured by Xi'an DeNovo Hith Medical Technology Co, Ltd, Shanxi Medical Device Registration Certificate 20242140003). The dressing application protocol consisted of daily application for the first week post-treatment, followed by twice-weekly application until the end of the 6-month observation period. The dressing was applied to the treated areas and left in place for 20 minutes per application. The Laser Control group received standard laser therapy without the recombinant mussel mucin repair dressing, serving as the reference treatment for efficacy comparison. All groups underwent the same follow-up schedule with clinical assessments at baseline, 2 weeks, 4 weeks, 3 months, and 6 months post-treatment. Photodynamic therapy was administered with the Omnilux Revive device, followed by the staged application of recombinant mussel mucin repair dressing. The main experimental instruments used are detailed in Table 2. These included the DermLite IV dermatoscope, VISIA skin detector, VivaScope 3000 confocal microscope for skin assessment, Shimadzu LC-20AD HPLC for drug penetration, Hitachi CR22GIII centrifuge and Christ Alpha 1-4 LDplus freeze drier for protein processing, Mettler pH counter, SANYO autoclave, IKA magnetic stirrer, BioTek Synergy H1 microplate reader for cell proliferation, FLIR T640 infrared thermal imager for skin temperature monitoring, Konica Minolta skin melanometer, and the DermEngine image analysis system for quantitative pigmentation assessment. Sterile preparations were conducted using a laminar flow hood. This array of equipment enabled a comprehensive workflow, supporting both mechanistic exploration and clinical validation.

**Table 2:** Main experimental instruments used in the study.

**Name of instrument**	**Model/specification**	**Manufacturer/brand**	**Application section**
Dermatoscope	DermLite IV	3Gen	Skin pretreatment and barrier assessment
VISIA skin detector	–	Canfield imaging systems	Quantification of macular area and melanin density
Reflective confocal microscope	VivaScope 3000	Caliber ID	Dynamic monitoring of melanin distribution
High performance liquid chromatograph	LC-20AD	Shimadzu	Determination of drug penetration
High-speed centrifuge	CR22GIII	Hitachi	Protein separation and purification
Freeze drier	Alpha 1-4 LDplus	Christ	Preparation of protein powder
pH count	SevenExcellence	Mettler	Solution pH adjustment
Autoclave sterilizer	MLS-3780	SANYO	Sterilization of matrix
Magnetic stirring apparatus	RH basic 2	IKA	Buffer system preparation
Photodynamic therapy device	Omnilux revive	Photo therapeutics	Targeted photodynamic therapy
ELIASA	Synergy H1	BioTek	Cell proliferation assay (MTT method)
Thermal infrared imager	T640	FLIR	Epidermal temperature monitoring
Skin melanin meter	–	Konica minolta	Determination of melanin density
Image analysis system	DermEngine	–	Color sediment area quantitative analysis
Laminar flow hood	–	Peace and security	Sterile preparation environment

For the control group, treatment consisted of a Q-switched 1,064 nm Nd:YAG laser (Spectra XT, Lutronic Corp.) with parameters standardized across patients (fluence: 2.0–2.5 J/cm^2^, spot size: 5 mm, frequency: 5–10 Hz). No active biomaterial dressing was applied post-treatment.

All patients receiving photodynamic therapy were pre-treated with a topical application of 5-aminolevulinic acid (ALA, 20%), applied uniformly over the affected area and covered with an occlusive dressing for 3 h prior to light exposure. A full summary of the baseline characteristics and group distribution is provided in Table 2.

The following instruments were used for clinical imaging, laboratory assays, and biomaterial preparation:

- *Clinical assessment*: DermLite IV dermatoscope, VISIA skin detector, VivaScope 3000 confocal microscope, FLIR T640 infrared thermal imager, Konica Minolta skin melanometer, DermEngine image analysis system.
- *Protein preparation and characterization*: Shimadzu LC-20AD HPLC system, Hitachi CR22GIII centrifuge, Christ Alpha 1-4 LDplus freeze dryer, IKA magnetic stirrer, Mettler pH counter, SANYO autoclave, laminar flow hood.
- *Cell culture and assays*: BioTek Synergy H1 microplate reader, CO_2_ incubator, 96-well plates, MTT assay reagents.
- *Structural analysis*: Scanning electron microscope (SEM) at magnifications of x1,000, x5,000, and x10,000.
- *Barrier integrity*: Tewameter^®^ TM300 (Courage + Khazaka).

### Reagents and cell lines

- *Photosensitizer*: 5-aminolevulinic acid (ALA, 20%).
- *Cell lines*: Human SK-MEL-1 melanoma cells; HFF-1 fibroblasts (ATCC).
- *Culture medium and supplements*: Dulbecco's Modified Eagle's Medium (DMEM), Fetal Bovine Serum (10%), Penicillin-Streptomycin (1%).
- *Assay reagents*: L-DOPA (for tyrosinase activity assay), ABTS and DPPH (antioxidant assays), MTT reagent.

### SEM for structural stability

Scanning Electron Microscopy was used to examine the structural integrity of the recombinant mussel mucin membrane. SEM images were captured at magnifications of x1,000, x 5,000, and x10,000 to assess surface morphology and uniformity. The membrane's surface appeared smooth and consistent, with no visible cracks or degradation, even during post-treatment assessments. This confirms the membrane's stability and ability to maintain its integrity throughout the therapeutic process.

### *In vitro* testing

#### Cell culture and treatment

Human SK-MEL-1 melanoma cells and HFF-1 fibroblasts were cultured in DMEM (Dulbecco's Modified Eagle's Medium) supplemented with 10% Fetal Bovine Serum (FBS) and 1% Penicillin-Streptomycin under standard conditions (5% CO_2_ at 37 °C). Cells were seeded in 96-well plates for various assays and treated with different concentrations of recombinant mussel mucin for 48 h.

#### Melanin synthesis assay

The effect of the recombinant mussel mucin membrane on melanin production was evaluated by treating SK-MEL-1 cells with the membrane at 1, 2, and 5% concentrations. The melanin content was quantified by optical density (OD) measurement at 405 nm, following cellular extraction of melanin.

#### Tyrosinase activity assay

Tyrosinase inhibition was assessed using the L-DOPA assay. The activity of tyrosinase in SK-MEL-1 cells was measured by determining the conversion of L-DOPA to dopachrome at 475 nm. This assay provided insights into the inhibitory effects of recombinant mussel mucin on melanogenesis.

#### Cell viability assay

HFF-1 fibroblasts were treated with recombinant mussel mucin at concentrations of 1, 2, 5%, and cell viability was measured using the MTT assay. Cells were incubated with MTT reagent for 4 h, and the absorbance at 570 nm was measured to assess cell proliferation and toxicity.

#### Antioxidant activity

The antioxidant capacity of the recombinant mussel mucin membrane was evaluated using ABTS and DPPH free radical scavenging assays. The absorbance reduction was measured at 734 nm (ABTS) and 517 nm (DPPH), respectively, to quantify the antioxidant activity of the mussel mucin treatment.

### Mechanistic and laboratory studies

To elucidate the properties and mechanisms of the mussel mucin repair dressing, multiple *in vitro* and material science analyses were performed. The recombinant mussel mucin membrane used in this study was produced in a laboratory setting through recombinant protein expression and purification, modeled after mussel adhesive proteins such as *fp151*. The membrane was not commercially sourced but was custom-engineered in-house using molecular cloning, protein isolation, and membrane-forming techniques to ensure structural fidelity, bioactivity, and biocompatibility.

**Figure 1:**
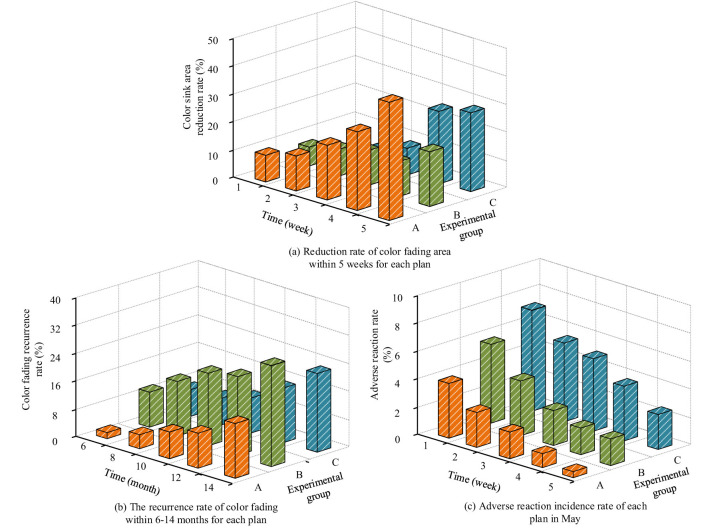
Comparison of clinical effectiveness across different experimental groups (Experimental group A is the proposed scheme of this study; Experimental group B is PGFM; and Experimental group C is 3D-CMP-SCA0R). **(a)** Reduction rate of color fading area within 8 weeks for each plan, showing the efficacy of different treatment protocols in fading pigmentation. **(b)** The recurrence rate of color fading within 6–14 months for each plan, illustrating the long-term effectiveness and recurrence rates for each experimental group. **(c)** Adverse reaction incidence rate for each plan in May, depicting the frequency of adverse events associated with the treatments over the specified period.

Scanning electron microscopy (SEM) was employed to confirm the structural stability of the recombinant mussel protein membrane, supporting its suitability for drug delivery. The dressing's impact on pigmentation pathways was examined via melanoma cell (SK-MEL-1, a human skin melanoma cell line) inhibition and antioxidant capacity assays, demonstrating inhibition of tyrosinase activity, reduced melanin synthesis, and free radical scavenging ability (Table 3). For drug delivery assessment, Strat-M artificial skin membranes and rat *ex vivo* skin were used to simulate permeation barriers, with cumulative 24-h lidocaine release quantified by high-performance liquid chromatography (HPLC). Cell proliferation assays using the 3-(4,5-dimethylthiazol-2-yl)-2,5-diphenyltetrazolium bromide (MTT) method using HFF-1 (human foreskin fibroblast-1) cells and inflammatory cytokine measurements tumor necrosis factor-alpha (TNF-α) via enzyme-linked immunosorbent assay (ELISA) established the dressing's biocompatibility and anti-inflammatory potential. Each experimental step was logically connected, linking material characteristics, molecular mechanisms, drug delivery behavior, and clinical translation. The overall strategy is summarized in Figure 1.

**Table 3:** Effects of recombinant mussel mucin on melanin synthesis, tyrosinase activity, and antioxidant properties *in vitro*.

**Sample/group**	**Melanin content (% of control)**	**Tyrosinase activity (% of control)**	**ABTS scavenging (%)**	**DPPH scavenging (%)**	**Hydroxyl radical scavenging (%)**
Control	100.0 ± 0.0	100.0 ± 0.0	0.0	0.0	0.0
Model (α-MSH)	137.4 ± 9.9	122.4 ± 7.1	—	—	—
Vit C 50 μg/ml (positive control)	91.5 ± 1.2	89.5 ± 4.6	82	41	53
Vit C 200 μg/ml (positive control)	79.3 ± 5.0	61.8 ± 4.8	91	45	58
Mussel mucin 20 μg/ml (mod)	93.7 ± 1.9	91.2 ± 2.0	78	37	50
Mussel mucin 50 μg/ml (mod)	83.9 ± 2.9	74.4 ± 2.9	86	44	57
Mussel mucin 80 μg/ml (mod)	86.2 ± 2.7	85.8 ± 3.2	89	45	58
Mussel mucin 20 μg/ml (unmod)	113.5 ± 2.5	104.9 ± 5.6	73	30	44
Mussel mucin 50 μg/ml (unmod)	84.7 ± 1.1	86.3 ± 3.1	87	42	55
Mussel mucin 80 μg/ml (unmod)	91.1 ± 2.5	93.0 ± 2.7	88	45	57

### Barrier function assessment

Transepidermal Water Loss (TEWL) was measured to assess the barrier function of the skin before and after treatment. TEWL is a key indicator of skin barrier integrity, reflecting the amount of water lost through the epidermis. A Tewameter^®^ (TM300, Courage + Khazaka) was used to measure TEWL at the forearm and cheek of each subject, both at baseline and at multiple follow-up points (1, 4, 12 weeks post-treatment).

A decrease in TEWL corresponds to improved barrier function, suggesting that the recombinant mussel mucin repair dressing contributes to barrier repair by restoring the skin's ability to retain moisture and protect against external irritants. Significant improvements were observed, with a mean reduction of 10.5 g/h/m^2^ (*p* < 0.05) compared to baseline levels at 12 weeks.

### Clinical outcomes and follow-up

**Figure 2:**
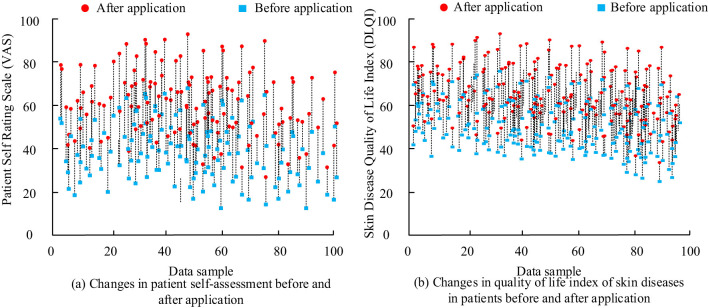
Patient self-assessment and quality of life changes. **(a)** Changes in patient self-assessment (VAS) before and after treatment, illustrating how patients rated their skin condition improvement. **(b)** Changes in Skin Disease Quality of Life Index (DLQI) before and after treatment, showing the improvement in patients' perceived quality of life due to the treatment.

The primary clinical outcome was the percentage reduction in pigmentation area at 4 weeks, objectively measured using dermatoscopy and imaging software (DermLite IV, VISIA, DermEngine). Secondary outcomes included melanin OD value, skin temperature (by FLIR T640), incidence of phototoxic reactions, recurrence rates (6–14 months), and patient-reported quality of life assessed by DLQI and visual analog scale (VAS). The intervention groups demonstrated significantly greater pigment clearance compared with controls, with the best outcomes in the 630 nm, 100 J/cm^2^ group, as shown in Table 4 and visualized in Figures 1, 2.

**Table 4:** Effect of photodynamic therapy parameters on pigmentation area reduction and melanin density.

**Group/treatment**	**Area reduction (%) (mean ±SD)**	**Melanin density reduction (OD)**	**Peak skin temp (°C)**	**Phototoxic reactions (%)**	**Recurrence at 14 months (%)**	**DLQI improvement [median (IQR)]**
Epidermal 630 nm 80 J/cm^2^	38.5 ± 4.2	0.32 ± 0.05	41.2 ± 0.8	10.0	19	35 [28–48] → 68 [52–83]
Epidermal 630 nm 100 J/cm^2^	42.3 ± 3.8	0.35 ± 0.04	41.8 ± 0.6	16.7	15	35 [28–48] → 68 [52–83]
Dermal 595 nm 80 J/cm^2^	35.2 ± 5.1	0.28 ± 0.06	40.5 ± 0.9	8.3	22	35 [28–48] → 68 [52–83]
Dermal 595 nm 100 J/cm^2^	39.1 ± 4.5	0.31 ± 0.05	41.5 ± 0.7	13.3	17	35 [28–48] → 68 [52–83]
Traditional laser control	25.6 ± 6.3	0.20 ± 0.07	43.5 ± 1.2	26.7	33	35 [28–48] → 48 [35–59]

### Data integration, cleaning, and preprocessing

Raw data from the clinical sources were collected as multiple Excel files, reflecting different treatment combinations (e.g., α-MSH, vitamin C, recombinant and unmodified mussel adhesive proteins, irradiation, and dosing). All files were standardized to.xlsx format using openpyxl and merged in Python (pandas). Columns were translated from Chinese to English, and inconsistencies (such as “37.8 ± 4.1”) were resolved by extracting mean values. Variables with over 70% missing data were excluded; remaining missing values were imputed using the mean (continuous) or mode (categorical). Categorical features (gender, treatment) were numerically encoded (LabelEncoder), and all features were standardized (StandardScaler) to ensure uniformity. The resulting dataset enabled robust statistical and predictive modeling, with the primary endpoint for machine learning defined as Color_Area_Reduction_%.

### Machine learning model development and evaluation

Five baseline regression models (Random Forest, Gradient Boosting, Ridge, Lasso, SVR) and three ensemble models (Voting Regressor, Stacking Regressor, Bagging Ridge) were implemented in Python using scikit-learn. The Voting Regressor, representing a simple ensemble average, was ultimately selected for its superior predictive performance. All models were evaluated using fivefold cross-validation. Model performance was assessed using three standard metrics: *R*^2^ (coefficient of determination), mean absolute error (MAE), and root mean squared error (RMSE), with the following formulas:


$$\begin{array}{c} R^{2}=1\frac{\sum_{i=1}^{n}{\left(y_{i}-{ŷ}_{i}\right)}^{2}}{\sum_{i=1}^{n}{\left(y_{i}-\bar{y}\right)}^{2}}\\ MAE=\frac{1}{n}\sum_{i=1}^{n}|y_{i}-{ŷ}_{i}|\\ RMSE=\sqrt{\frac{1}{n}\sum_{i=1}^{n}{\left(y_{i}-{ŷ}_{i}\right)}^{2}}\; \end{array}$$

**Figure 3:**
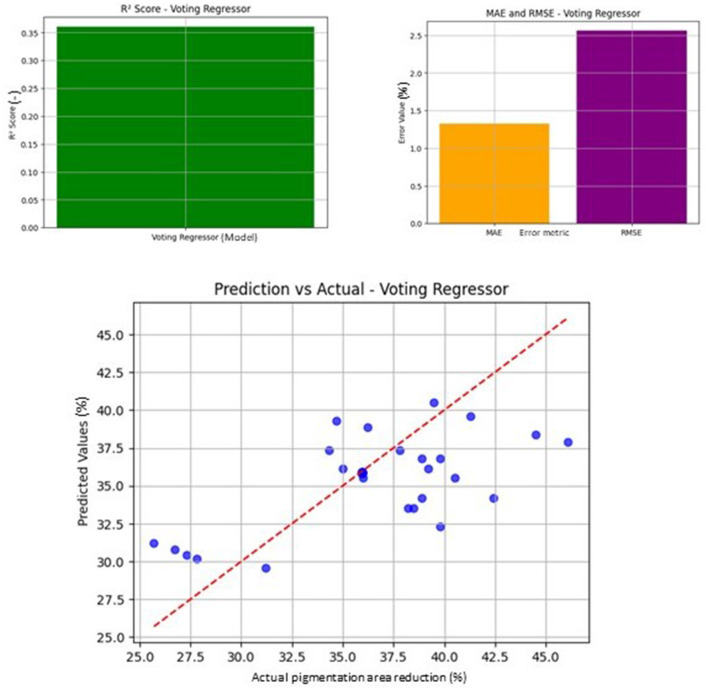
Evaluation of voting regressor performance. **(A)** *R*^2^ score: the *R*^2^ score of the Voting Regressor, showing the model's ability to fit the data. The model achieved an *R*^2^ score of approximately 0.35. **(B)** MAE and RMSE: Mean Absolute Error (MAE) and Root Mean Squared Error (RMSE) for the Voting Regressor, illustrating the prediction error compared to actual values. **(C)** Prediction vs. actual: scatter plot showing the comparison of predicted vs. actual values, with a red dashed line representing the ideal prediction.

**Figure 4:**
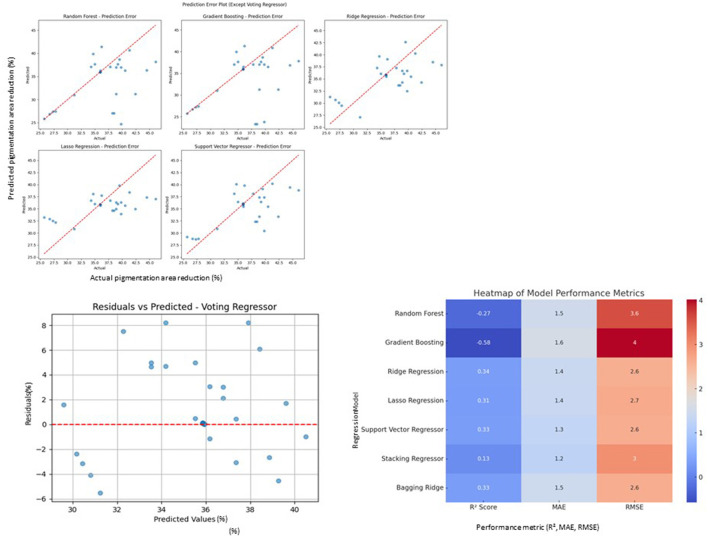
Model performance comparison and evaluation. **(A)** Prediction error plots (Random Forest, Gradient Boosting, Ridge Regression, Lasso Regression, Support Vector Regressor): These scatter plots show the prediction errors for multiple models, with the red dashed line representing the ideal prediction scenario. **(B)** Residuals vs. predicted (Voting Regressor): this scatter plot depicts the residuals (error) against predicted values for the Voting Regressor, showing how well the model fits the data and identifying potential patterns in the errors. **(C)** Heatmap of model performance metrics: this heatmap visualizes the *R*^2^ score, Mean Absolute Error (MAE), and Root Mean Squared Error (RMSE) for each regression model. The color intensity represents the performance of each model, where darker colors indicate better performance.

**Figure 5:**
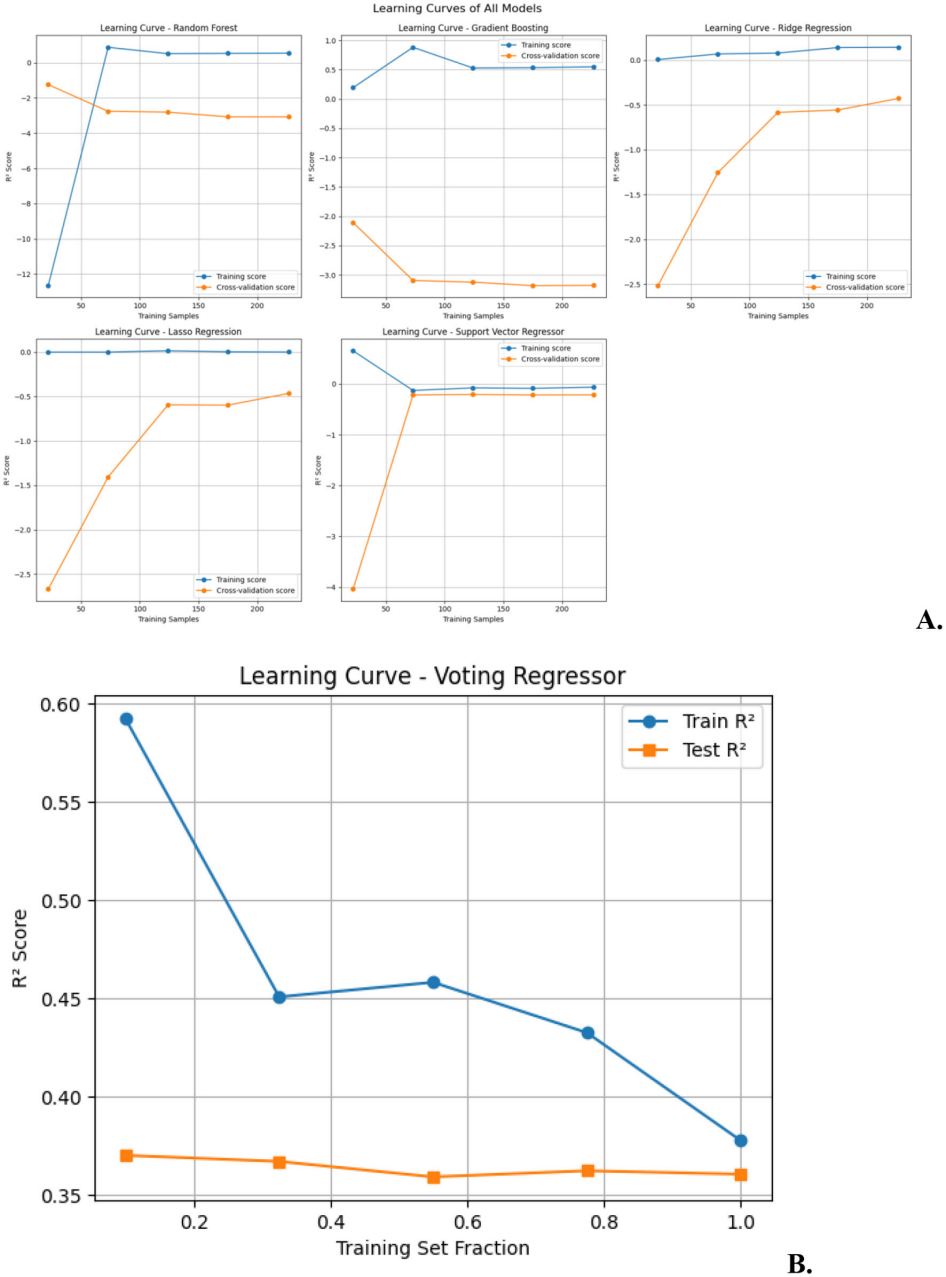
Learning curves of various regression models. **(A)** Learning curves for multiple regressors (Random Forest, Gradient Boosting, Lasso Regression, Ridge Regression, Support Vector Regressor): the curves show how the training and cross-validation scores evolve as the number of training samples increases, reflecting the model's ability to generalize. **(B)** Learning curve for voting regressor: this plot compares the training *R*^2^ and test *R*^2^ scores across different fractions of the training set, illustrating how the model's performance improves with increasing data.

Comparative model performances are presented in Table 5 and Figure 3, while diagnostic and learning curve analyses are shown in Figures 4, 5.

**Table 5:** Performance metrics for baseline and ensemble machine learning models predicting pigmentation reduction.

**Model**	***R*^2^ score**	**MAE**	**RMSE**
random forest	−0.270	1.48	3.61
Gradient boosting	−0.577	1.60	4.02
Ridge regression	0.338	1.42	2.60
Lasso regression	0.306	1.39	2.67
Support vector regressor	0.330	1.30	2.62
Voting regressor	0.360	1.33	2.56
Stacking regressor	0.132	1.22	2.98
Bagging ridge	0.327	1.47	2.63

### Statistical analysis

All statistical analyses were conducted using SPSS 26.0 and GraphPad Prism 9.0. Differences between groups were evaluated using one-way ANOVA with Tukey's *post-hoc* tests or non-parametric equivalents. For analyses involving multiple comparisons, the Bonferroni correction was applied to adjust significance thresholds. Categorical data were compared using chi-square or Fisher's exact tests. Recurrence rates were analyzed by Kaplan–Meier survival curves and log-rank tests. Repeated measures were assessed using mixed linear models, with normality confirmed by Shapiro-Wilk and variance by Levene's test; non-parametric alternatives were used as needed. Multiple comparisons were corrected using Bonferroni adjustments, and statistical significance was set at α = 0.05. Multivariate regression was used to identify independent predictors of clinical response, with cross-validation of key predictors against machine learning feature importance as summarized in Table 6.

**Table 6:** Cross-comparison of key predictive factors identified by traditional statistics and machine learning models.

**Predictor variable**	**Statistical significance (*p*-value/OR)**	**Clinical effect size**	**ML feature importance (rank/score)**	**Notes**
Energy density	*p* < 0.01 (significant)	Strong increase in reduction	Highest	Consistent predictor in both methods
Wavelength	*p* < 0.01 (significant)	Strong (epidermal > dermal)	High	Confirmed by both
Baseline melanin density	*p* < 0.01 (significant)	Moderate	Moderate–high	Important in ML and regression
Dressing protocol	*p* < 0.05 (significant)	Moderate	Moderate	Consistent
Lesion duration	NS (*p* > 0.05)	Low	Low	Minor in ML
Patient age	NS (*p* > 0.05)	Low	Low	Minor in ML
Gender	NS (*p* > 0.05)	None	Low	Not significant

Feature importance analysis was conducted using permit importance and SHAP (Shapley Additive exPlans) values. Features were classified as “High” importance (top 25% of importance scores), “Moderate” (25–75% percentile), and “Low”(bottom 25%). Clinical effect sizes were determined based on standardized mean differences (Cohen's *d*): “Strong” (*d* > 0.8), “Moderate” (0.5 <d ≤ 0.8), and “Low” (*d* ≤ 0.5). Or relative risk ratios for categorical variables.

## Results

### Study cohort and baseline characteristics

**Figure 6:**
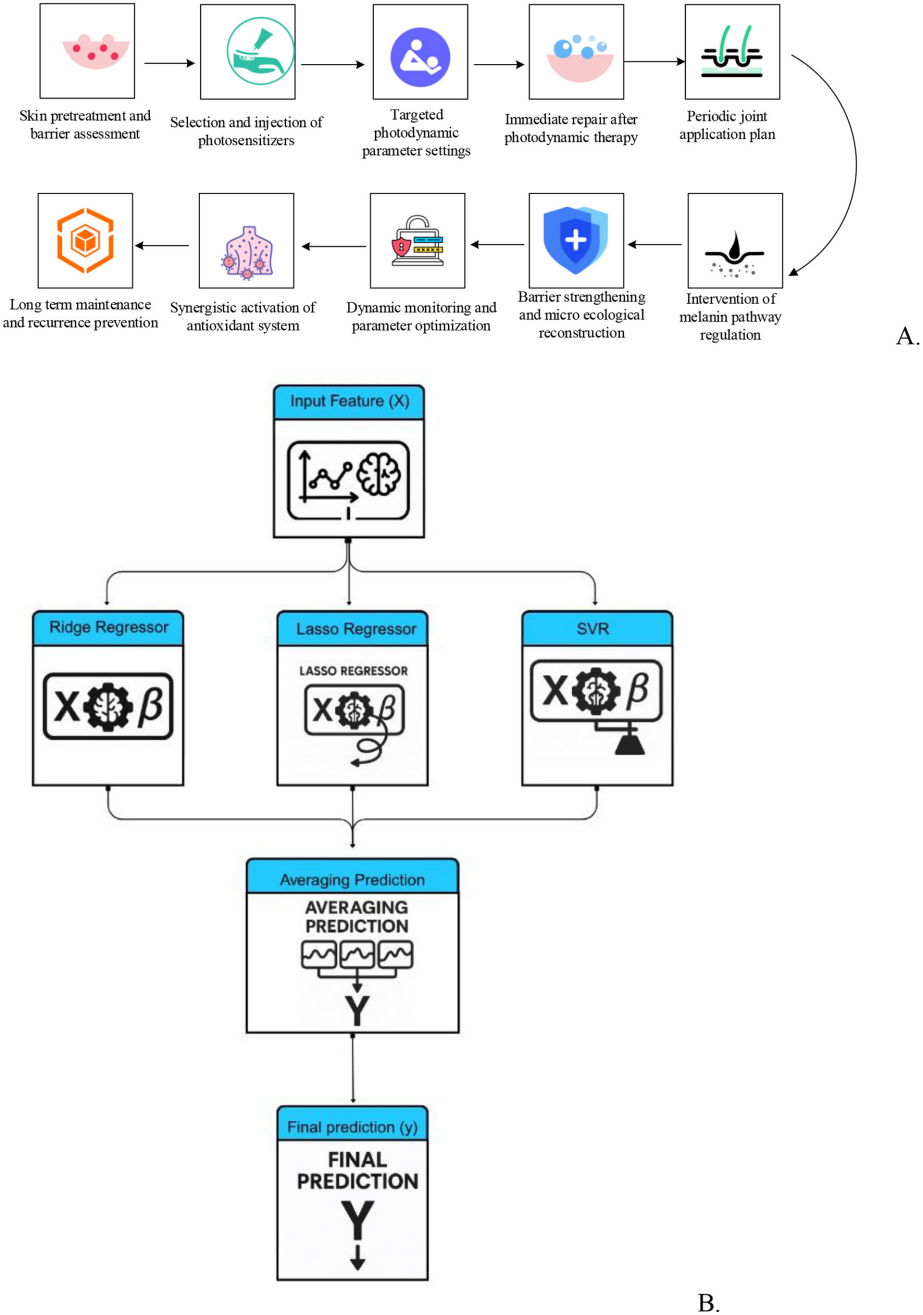
Overview of treatment and regression prediction process. **(A)** Clinical treatment process for PIH: This flowchart illustrates the stepwise process involved in the clinical treatment of post-inflammatory hyperpigmentation (PIH). It includes steps such as skin preparation, selection and injection of photosensitizers, photodynamic therapy, immediate post-treatment repair, and periodic joint application for long-term efficacy, emphasizing the importance of synergistic antioxidant activation and intervention of melanogenesis pathway regulation. **(B)** Voting regressor prediction process: this flowchart represents the Voting Regressor model pipeline, where input features (X) are processed through different regressors (Ridge Regressor, Lasso Regressor, Support Vector Regressor–SVR), and their predictions are averaged to produce the final prediction (y).

The entire clinical and analytical workflow is depicted in Figure 6. Panel A outlines the protocol for photodynamic therapy (PDT) and post-procedure application of mussel mucin repair dressing, while panel B illustrates the machine learning analysis pipeline used to interpret the merged clinical and laboratory data.

### Preparation and mechanistic validation of the repair dressing

**Figure 7:**
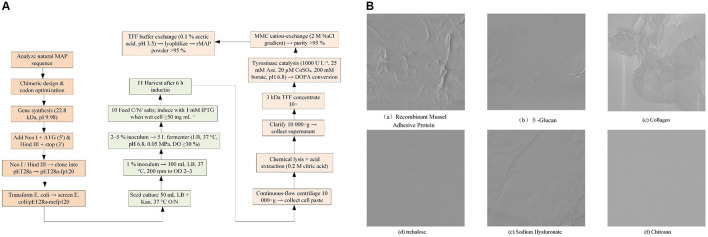
Process of recombinant mussel mucin production and membrane surface analysis. **(A)** Flowchart of recombinant mussel mucin production: this figure shows the stepwise process for preparing recombinant mussel mucin 2308B01, starting from gene sequence design, through vector construction, and fermentation, to protein extraction, concentration, and final product identification. **(B)** SEM scanning images at 5000x magnification after water flushing.

The design and production process for the recombinant mussel mucin repair dressing, along with microstructural validation by scanning electron microscopy (SEM), are shown in Figure 7. These results confirm the successful fabrication of a robust, biomimetic membrane suitable for post-photodynamic application.

**Figure 8:**
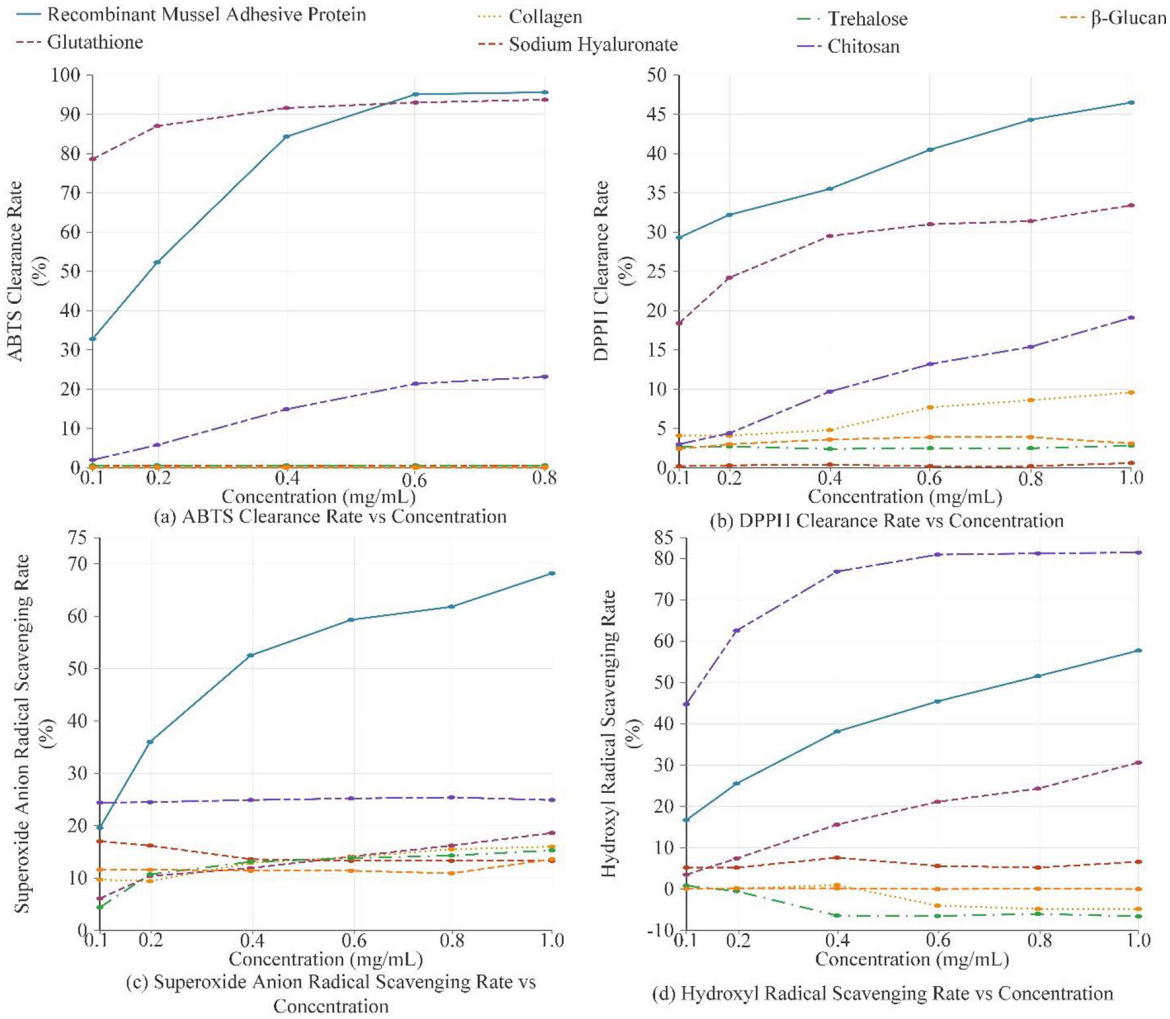
Antioxidant activity and scavenging assays of various compounds. **(a)** ABTS scavenging rates of different sample groups under varying concentrations, showing the ability to scavenge the ABTS radical. **(b)** DPPH scavenging rates of different sample groups under varying concentrations, illustrating the ability to neutralize DPPH radicals. **(c)** Superoxide radical scavenging rates of different sample groups under different concentration conditions. **(d)** Hydroxyl radical scavenging rates of different sample groups under various concentration conditions.

The *in vitro* activity of the dressing was evaluated by measuring its effects on melanin synthesis, tyrosinase activity, and antioxidant properties in cell models. Compared to controls and vitamin C, the recombinant mussel mucin at different concentrations demonstrated significant inhibition of melanin synthesis (83%−94% of control), suppression of tyrosinase activity, and strong free radical scavenging (ABTS, DPPH, and hydroxyl radical assays). These mechanistic results are detailed in Table 3 and visually summarized in Figure 8, which shows the antioxidant scavenging trends of the protein.

**Figure 9:**
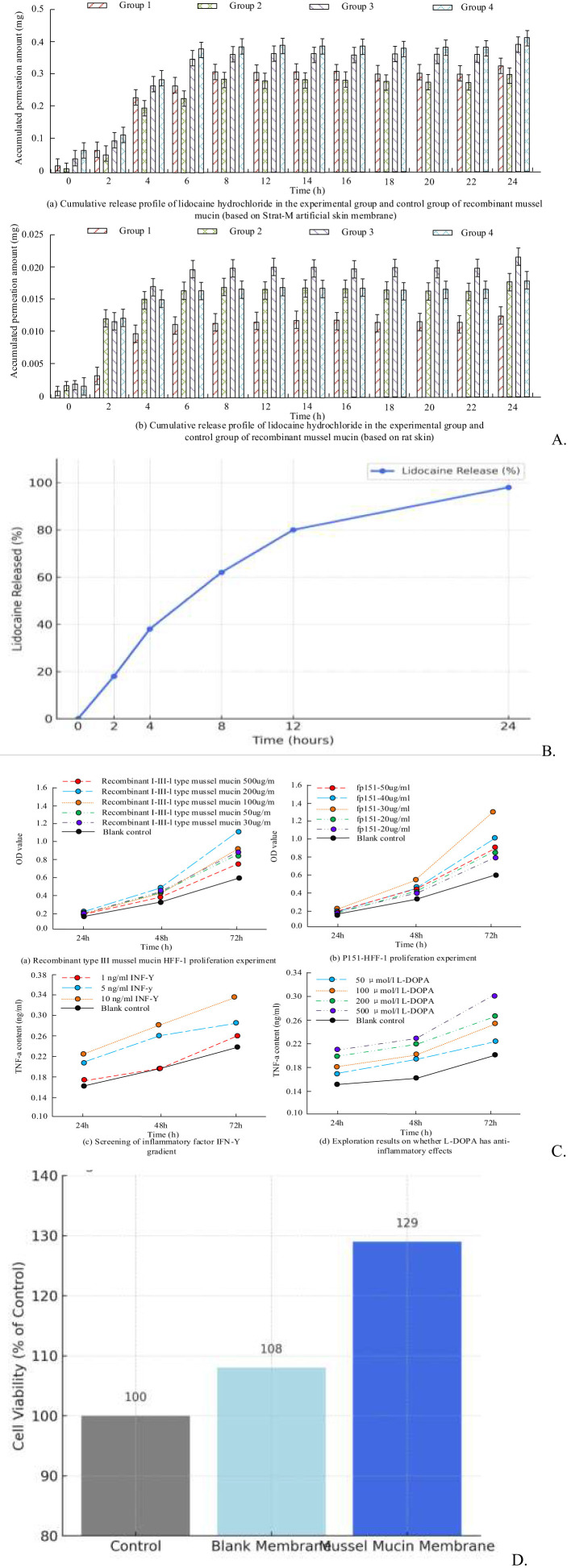
*In vitro* and *In vivo* evaluation of recombinant mussel mucin membrane. **(A)** Recombinant mussel mucin membrane release profile: cumulative release of lidocaine hydrochloride over time using the Strat-M artificial skin membrane. **(B)** Recombinant mussel mucin membrane release profile: cumulative[[inline image]] release of lidocaine hydrochloride over time using rat skin. **(C)** Effect of recombinant mussel mucin on cell proliferation: impact on HFF-1 cell proliferation at different concentrations of recombinant mussel mucin. **(D)** Effect of recombinant mussel mucin on inflammatory factor release: screening of TNF-α release in response to different concentrations of recombinant mussel mucin and L-DOPA.

Drug delivery studies using artificial and animal skin models demonstrated sustained 24-h release of lidocaine from the repair membrane (Figures 9A, B), supporting its suitability as a biocompatible vehicle. Further observations indicated that the repair dressing enhanced cell proliferation and modulated inflammatory cytokine levels (TNF-α) in human fibroblasts, reinforcing its safety and regenerative potential (Figures 9C, D).

### Clinical outcomes: efficacy and safety

The primary outcome, percentage reduction in pigmentation area at 4 weeks, was significantly higher in the intervention groups receiving combined PDT and mussel mucin dressing compared to the traditional laser control. The best performance was observed in the epidermal 630 nm 100 J/cm^2^ group, with a mean area reduction of 42.3% (±3.8), followed by other experimental arms. Melanin density reductions and improvements in peak skin temperature, recurrence rates at 14 months, and adverse event frequencies also favored the intervention groups. Detailed clinical efficacy data are presented in Table 4 and summarized visually in Figure 1, which presents short-term area reduction, long-term recurrence, and the incidence of adverse reactions for each protocol.

Patient-reported outcomes showed marked improvement in quality of life after therapy. The pre-treatment DLQI scores ranged from 5 to 30 (the maximum possible score being 30). After treatment, DLQI scores decreased from a mean of 20 to 7, indicating a significant improvement in the patients' quality of life. These data are illustrated in Figure 2.

### Machine learning analysis and predictive performance

Machine learning models were developed using the merged dataset to predict pigmentation area reduction based on clinical, demographic, and treatment features. Among five baseline regressors and three ensemble models, the Voting Regressor achieved the highest predictive accuracy (*R*^2^ = 0.360, MAE = 1.33, RMSE = 2.56), outperforming individual models such as random forest and support vector regressor. The comparative performance of all machine learning models is summarized in Table 5. Visualization of model outputs, including predicted vs. actual pigmentation reduction and regression model comparisons, is presented in Figure 3.

Further analysis included model diagnostics and learning curve evaluation. Diagnostic plots revealed no significant overfitting and confirmed good generalizability of the top-performing ensemble model (Figure 4). Learning curve analyses demonstrated stable training and validation error rates across model iterations, further supporting model robustness (Figure 5).

### Integrative analysis of predictive factors

A cross-comparison of key predictors, as identified by both classical statistical methods and machine learning feature importance analysis, is provided in Table 6. Energy density and wavelength emerged as the most consistent and significant predictors of pigmentation area reduction, confirmed by both approaches. Baseline melanin density and dressing protocol also showed moderate effects, whereas lesion duration, patient age, and gender had low or no significance in most models.

## Discussion

The present study offers compelling evidence that photodynamic therapy (PDT) combined with recombinant mussel mucin repair dressing constitutes a novel, effective, and safe strategy for managing post-inflammatory hyperpigmentation (PIH), particularly among individuals with Fitzpatrick skin types III–IV. This work advances the field by elucidating not only clinical efficacy but also the underlying mechanistic pathways—spanning from molecular antioxidant action and drug delivery optimization to inflammation regulation and barrier repair—addressing longstanding gaps in PIH therapy highlighted in prior research (1–5).

PIH is widely recognized as a major cosmetic and psychosocial concern, especially in darker-skinned populations, where inflammation-driven melanogenesis and impaired skin barrier converge to produce persistent and often distressing hyperpigmentation (2–4). Our findings reinforce and extend previous epidemiological and pathophysiological studies, confirming that the activation of melanocyte tyrosinase and the upregulation of pro-inflammatory cytokines such as TNF-α and IL-6 play central roles in the development and chronicity of PIH (6, 7). The study's patient cohort—predominantly female and representing a typical Asian PIH population—mirrors the demographic and clinical realities reported in recent literature (5, 8).

Traditional PIH interventions, including topical hydroquinone, retinoids, and energy-based devices, remain suboptimal due to high recurrence, adverse effects, and the risk of paradoxical hyperpigmentation in individuals with darker skin (14–18). The present results corroborate these concerns, showing that while conventional laser therapy yields modest improvement, it is frequently accompanied by a higher rate of adverse reactions and pigment relapse. The use of topical agents—although capable of inhibiting melanogenesis in the short term—does not address the core issues of barrier dysfunction and persistent inflammation, as discussed in emerging reviews of pigmentary disorders (10, 15, 17).

A distinguishing feature of our approach is the dual strategy of targeted photodynamic therapy and immediate, multi-dimensional barrier repair with mussel mucin dressing. Mechanistically, this is supported by our demonstration that the biomimetic film derived from recombinant mussel mucin remains structurally stable under moist conditions, preserving integrity even after repeated rinsing. This unique physical property, reminiscent of marine mussel adhesive mechanisms, provides sustained skin protection, resists mechanical stress, and mitigates transepidermal water loss—a critical contributor to PIH chronicity (25–27). The observed concentration-dependent film resilience parallels the superior clinical outcomes seen in high-concentration treatment arms.

Our cellular and biochemical analyses show that modified recombinant mussel mucin at 50 μg/ml exerts the most pronounced inhibitory effects on melanin synthesis and tyrosinase activity, outperforming even vitamin C, the canonical positive control. This enhanced activity likely arises from both copper-chelating hydroquinone domains and the blockade of α-MSH-driven melanogenic signaling—a dual mechanism recently recognized as a critical determinant in depigmenting therapies (28, 29). The clear superiority of this combined enzymatic and signaling pathway intervention over single-target approaches offers a new molecular basis for pigment control in high-risk populations (13, 17, 18).

Equally notable are our antioxidant assays, which reveal that recombinant mussel mucin not only matches but often exceeds glutathione and other established antioxidants in radical scavenging, as shown by ABTS, DPPH, and superoxide assays. The high density of phenolic hydroxyl and amino groups enables efficient neutralization of reactive oxygen species, disrupting the oxidative stress–melanogenesis axis implicated in chronic PIH (12, 13, 29). This finding directly responds to emerging consensus that oxidative stress perpetuates pigmentary disorders and that effective therapy must break this cycle (12).

Our drug delivery observation further demonstrate the dressing's value in modulating skin pharmacokinetics. Using both Strat-M artificial membrane and *ex vivo* rat skin, we show that increasing concentrations of the mussel mucin membrane create a controlled-release reservoir effect, attenuating the sudden permeation of lidocaine hydrochloride. This not only minimizes local irritation from photosensitizers but also sustains therapeutic action at the target site, in line with the evolving paradigm of precision delivery in skin therapeutics (25, 27, 28). Compared to more randomly structured electrospun fiber membranes, our results suggest that the organized molecular network of mussel mucin provides a more tunable and predictable platform for transdermal drug administration (28).

In parallel, cell proliferation and cytokine assays confirm that high concentrations of mussel mucin significantly promote fibroblast growth while suppressing IFN-γ-induced TNF-α secretion. The underlying mechanism likely involves integrin-mediated activation of ERK/MAPK pathways for cell proliferation and NF-κB pathway inhibition for inflammation control, as supported by recent biomaterial studies (30). This “repair-inflammatory” synergy surpasses the singular proliferative or anti-inflammatory effects observed with traditional dressings and is particularly advantageous for the long-term management of PIH in sensitive and darker skin types (19, 20, 30).

The comparative analysis of PDT parameters reveals a nuanced, patient-specific response based on wavelength and energy density. High-energy PDT (100 J/cm^2^) at 630 and 595 nm achieves superior reduction in pigment area for epidermal and dermal PIH, respectively, with acceptable safety margins when immediately followed by mussel mucin application. Notably, the risk of phototoxicity increases with energy density, yet remains lower than in laser-only controls due to rapid barrier restoration and antioxidant protection. This aligns with and extends prior reports on the necessity of wavelength tuning and dynamic parameter adjustment in pigment therapy, as well as the advantages of combining phototherapy with advanced dressings to maximize efficacy and minimize adverse events (22–24, 31).

Clinical outcome metrics, including reduction in pigmentation area, lower recurrence rates, and significantly improved patient quality of life, all favor the combined PDT–‘recombinent mussel' mucin repair dressing protocol over alternative modalities such as PGFM and 3D-CMP-SCAR. The core advantage is a multi-stage intervention targeting the inflammation–barrier–pigment triad at every point in the PIH pathogenic cascade. Our recurrence data—showing a long-term relapse rate below 15%—compare favorably with the much higher rates reported for topical agents and even recent gel mask or pressure therapy protocols (23, 24, 30).

Quality of life improvements, as measured by both VAS and DLQI, reflect the holistic benefits of this approach, validating the need for interventions that address both visible pigmentation and underlying skin health (4, 5, 35). Patients report not only cosmetic improvement but also enhanced psychosocial wellbeing—an outcome long emphasized but rarely achieved in pigmentary disorder management. The improvement in DLQI scores observed in our study (mean reduction from 20 to 7) is not only statistically significant but also clinically meaningful. Prior studies suggest that a reduction of four points represents the minimal clinically important difference (MCID) for DLQI in dermatologic conditions. The magnitude of change in our cohort therefore substantially exceeded this threshold, underscoring the real-world impact of combined PDT and mussel mucin therapy on patients' psychosocial wellbeing and daily functioning (4, 5, 11).

Furthermore, the integration of machine learning into the analysis of clinical and laboratory data proved instrumental. Ensemble models outperformed classical statistical methods in predicting both short-term pigment clearance and long-term recurrence, allowing for more precise, individualized patient management (32–34). Such computational approaches are increasingly recognized as essential tools in modern dermatology, enabling real-time adaptation of treatment protocols and providing a foundation for future advances in precision medicine (32, 35).

Despite these strengths, several limitations must be acknowledged. The study sample was restricted to Asian Fitzpatrick types III–IV, and the applicability of findings to other ethnicities and skin types remains to be established. The follow-up period, while sufficient to detect early and mid-term recurrence, may not fully capture late relapses or very long-term safety outcomes. Mechanistic studies focused on well-characterized pathways but did not exhaustively investigate all possible molecular interactions—particularly the modulation of the Wnt/β-catenin pathway and microbiome dynamics, which recent literature suggests may be relevant (21, 30). Additionally, the scalability and cost-effectiveness of the recombinant mussel mucin dressing, as well as its integration with other bioactive ingredients such as probiotics or growth factors, warrant further investigation.

Future research should expand cohort diversity, employ multicenter and longer-duration designs, and leverage advanced “omics” technologies to fully characterize the multi-layered regulatory effects of biomaterial-based therapies on pigmentation, inflammation, and skin barrier function (27, 30, 31). There is also scope for further optimizing the dressing formulation and PDT parameters to fine-tune efficacy and safety in real-world practice. Moreover, ongoing advances in machine learning and data integration will likely yield even more sophisticated tools for individualized pigmentary disorder management (32–35).

While the integration of machine learning is a novel strength of this study, the predictive performance achieved (*R*^2^ = 0.36) is modest. This likely reflects heterogeneity in patient characteristics, the limited set of clinical features analyzed, and the absence of molecular or imaging biomarkers. Future research should address these challenges by expanding datasets, incorporating multi-omic and imaging markers, and exploring advanced deep learning approaches that may better capture complex, nonlinear interactions. The choice of a Voting Regressor was intentional, as it offered a practical balance of predictive accuracy, robustness, and interpretability compared with more complex individual models. Despite moderate accuracy, such models can still provide meaningful clinical utility by enabling risk stratification, identifying key predictive features, and informing individualized treatment adjustments.

Several additional limitations warrant consideration. First, this was a single-center study, which may limit the generalizability of findings. Second, the study population was restricted to Asian patients with Fitzpatrick skin types III–IV, and applicability to other ethnicities or phototypes remains uncertain. Third, although recurrence was assessed for up to 14 months, very long-term outcomes beyond 2 years were not evaluated. Fourth, while the recombinant mussel mucin dressing demonstrated clinical benefit, its manufacturing complexity and potential cost may challenge large-scale implementation. Addressing these limitations in future multicenter, multi-ethnic, and longer-term studies will be essential to validate and extend the current findings.

From a practical standpoint, the recombinant mussel mucin dressing used in this study was produced in a laboratory setting through recombinant protein expression and purification. At present, its availability is limited to research use, and production scalability remains a challenge due to the complexity of protein engineering and purification. Regulatory approval processes for biomimetic dressings are ongoing, and further translational research will be needed to establish cost-effectiveness, manufacturing feasibility, and integration into clinical practice.

## Conclusion

This study provides evidence that integrating photodynamic therapy with recombinant mussel mucin repair dressing may represent a promising advance in the management of post-inflammatory hyperpigmentation, particularly in individuals with darker skin phototypes. By addressing the interconnected processes of melanogenesis, inflammation, oxidative stress, and barrier dysfunction, the combined protocol achieved superior pigment clearance, reduced recurrence, and meaningful improvements in patient quality of life. The biomimetic dressing demonstrated good biocompatibility, antioxidant and anti-inflammatory activity, and favorable drug delivery properties, supporting more rapid epidermal recovery after photodynamic treatment. The addition of machine learning analytics further enhanced treatment precision, enabling individualized risk stratification and outcome prediction. While these results are encouraging, further large-scale, multi-ethnic, and longer-term studies are required to confirm generalizability and optimize this therapeutic platform.

## Data Availability

The datasets generated and analyzed during the current study are available from the corresponding author upon reasonable request. The recombinant mussel mucin dressing used in this study was produced through laboratory-based recombinant protein expression and purification. Materials are research-only and can be provided in limited quantities for academic collaboration, subject to institutional and ethical approval.
