# Correction: Clinical efficacy and predictive factors of photodynamic therapy combined with recombinant mussel mucin repair dressing in treating post-inflammatory hyperpigmentation

**DOI:** 10.3389/fmed.2026.1831015

**Published:** 2026-06-24

**Authors:** Jianjun Nie, Zengmiao Hou, Peimei Zhou, Ya Zhang, Yonghong Lu

**Affiliations:** 1 Chengdu Second People's Hospital, Chengdu, China; 2 Xi an DeNovo Hith Medical Technology Co, Ltd, Xian, China; 3 The Eighth Affiliated Hospital of Sun Yat-sen University, Shenzhen, China

**Keywords:** post-inflammatory hyperpigmentation, photodynamic therapy, mussel mucin, repair dressing, machine learning, precision dermatology, melanogenesis, antioxidant therapy

There was a mistake in Figure 7 as published. Figures 7A and B were published without detailed labels identifying the chemical compounds used in the study. For improved clarity and completeness, the figures have been updated to include the names of the chemicals used. The figures themselves remain unchanged, and this correction does not affect the scientific results or conclusions of the article.

The corrected Figure 7 appears below.

**Figure 7:**
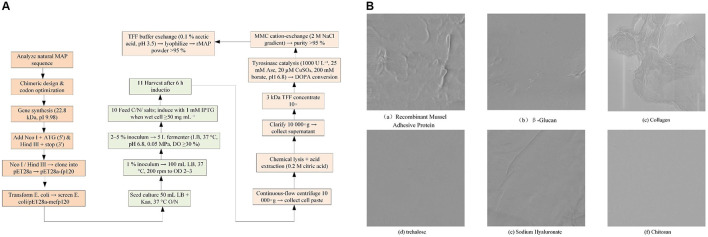
Process of recombinant mussel mucin production and membrane surface analysis. **(A)** Flowchart of recombinant mussel mucin production: this figure shows the stepwise process for preparing recombinant mussel mucin 2308B01, starting from gene sequence design, through vector construction, and fermentation, to protein extraction, concentration, and final product identification. **(B)** SEM scanning images at 5,000x magnification after water flushing.

There was a mistake in Figure 8 as published. Figure 8 was published without detailed labels identifying the chemical compounds used in the study. For improved clarity and completeness, the figures have been updated to include the names of the chemicals used. The figures themselves remain unchanged, and this correction does not affect the scientific results or conclusions of the article. The corrected Figure 8 appears below.

**Figure 8:**
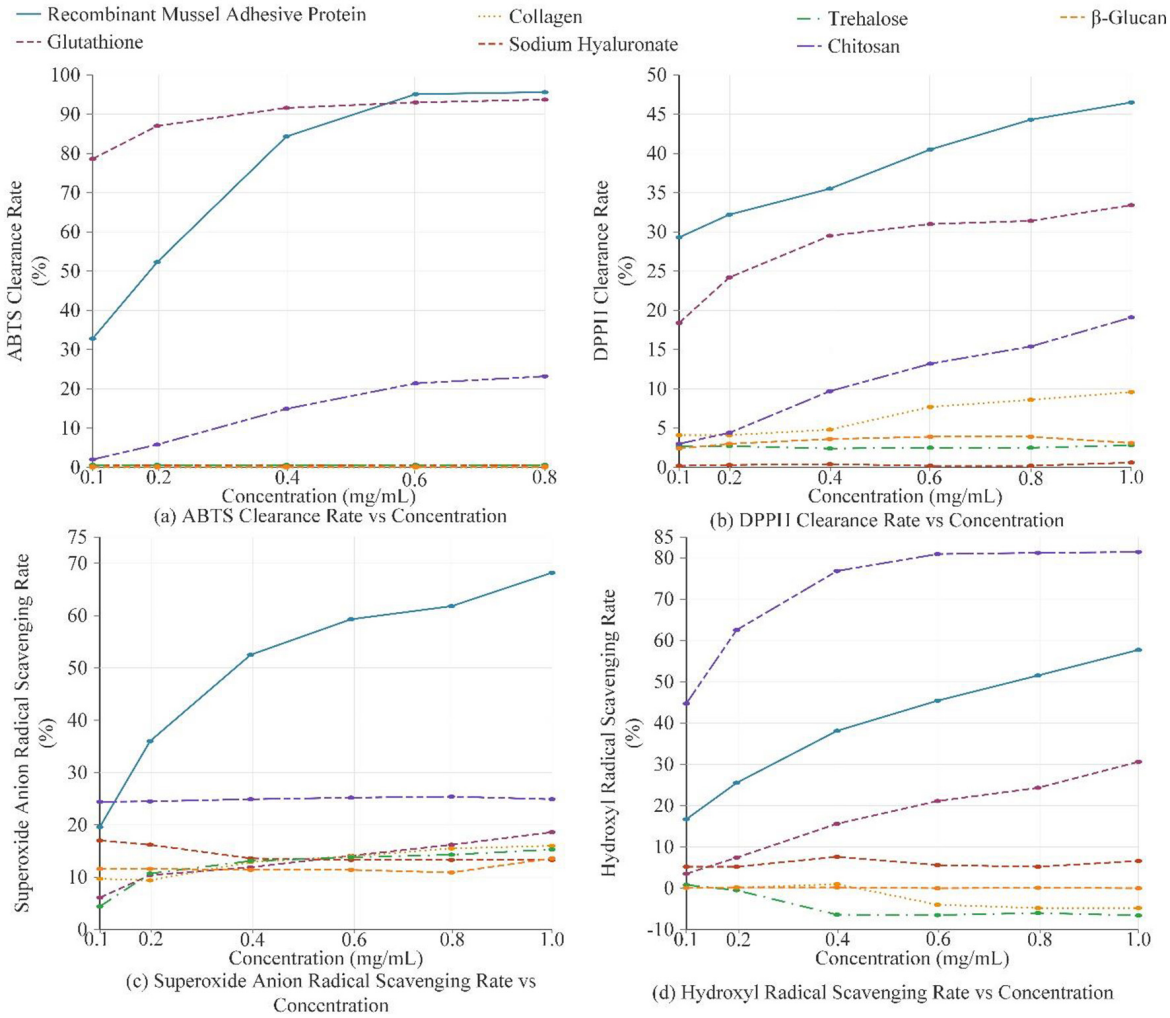
Antioxidant activity and scavenging assays of various compounds. **(a)** ABTS scavenging rates of different sample groups under varying concentrations, showing the ability to scavenge the ABTS radical. **(b)** DPPH scavenging rates of different sample groups under varying concentrations, illustrating the ability to neutralize DPPH radicals. **(c)** Superoxide radical scavenging rates of different sample groups under different concentration conditions. **(d)** Hydroxyl radical scavenging rates of different sample groups under various concentration conditions.

There was a mistake in Figure 9 as published. Figures 9A was published without detailed labels identifying the chemical compounds used in the study. For improved clarity and completeness, the figures have been updated to include the names of the chemicals used. The figures themselves remain unchanged, and this correction does not affect the scientific results or conclusions of the article. The corrected Figure 9 appears below.

**Figure 9:**
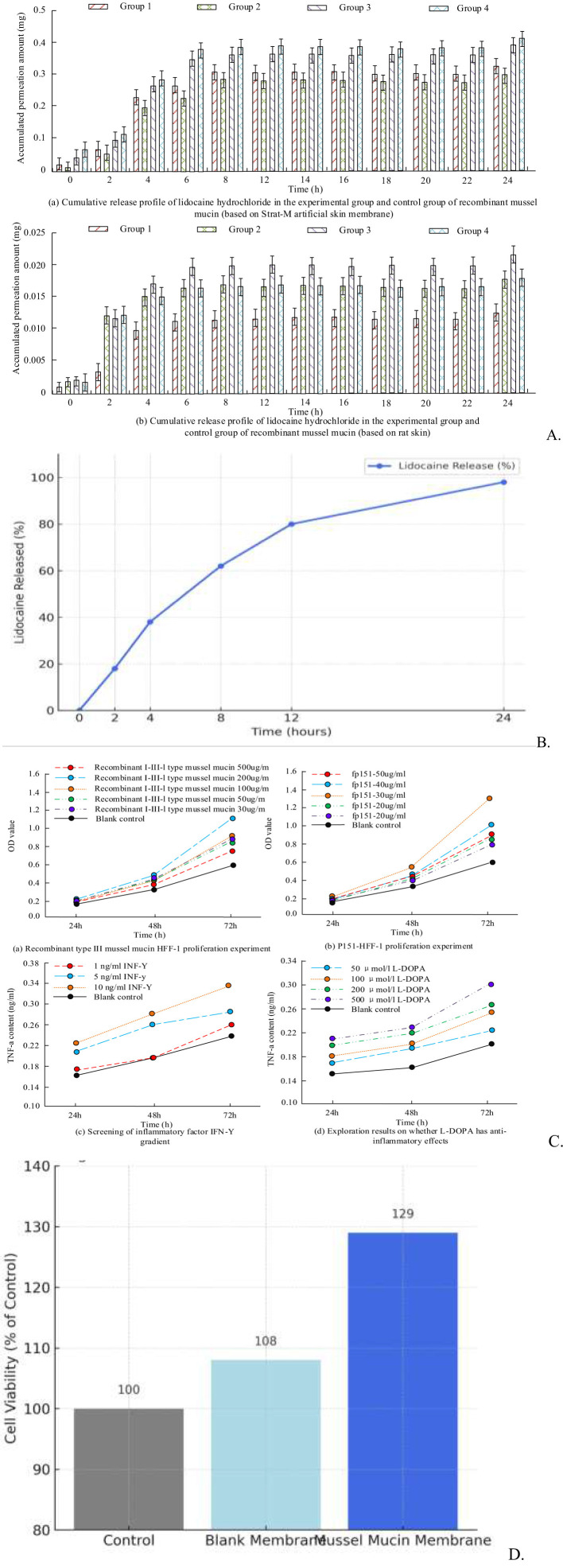
*In vitro* and *In vivo* evaluation of recombinant mussel mucin membrane. **(A)** Recombinant mussel mucin membrane release profile: cumulative release of lidocaine hydrochloride over time using the Strat-M artificial skin membrane. **(B)** Recombinant mussel mucin membrane release profile: cumulative[[inline image]] release of lidocaine hydrochloride over time using rat skin. **(C)** Effect of recombinant mussel mucin on cell proliferation: impact on HFF-1 cell proliferation at different concentrations of recombinant mussel mucin. **(D)** Effect of recombinant mussel mucin on inflammatory factor release: screening of TNF-α release in response to different concentrations of recombinant mussel mucin and L-DOPA.

The original version of this article has been updated.

